# Genomic characterization of noroviruses from an outbreak associated with oysters

**DOI:** 10.1128/spectrum.02580-24

**Published:** 2025-01-10

**Authors:** Annika Flint, Jennifer Harlow, Madison McLeod, Madeleine Blondin-Brosseau, Kelly Weedmark, Neda Nasheri

**Affiliations:** 1 Genomics Laboratory, Bureau of Microbial Hazards, Health Canada, Ottawa, Ontario, Canada; 2 National Food Virology Reference Center, Bureau of Microbial Hazards, Health Canada, Ottawa, Ontario, Canada; 3 Department of Biochemistry, Microbiology and Immunology, Faculty of Medicine, University of Ottawa, Ottawa, Ontario, Canada; US Food and Drug Administration, Silver Spring, Maryland, USA

**Keywords:** norovirus, outbreak, oysters, Illumina MiSeq, phylogenetic analysis, single nucleotide polymorphism

## Abstract

Human noroviruses are the leading cause of non-bacterial shellfish-associated gastroenteritis. In 2022, a multi-jurisdictional norovirus outbreak associated with contaminated oysters occurred that involved hundreds of illnesses. Here, we conducted genetic analysis on 30 clinical samples associated with this oyster outbreak. We first determined the capsid genotypes by Sanger sequencing and viral titers by droplet-digital reverse transcription PCR. Multiple genotypes were identified in this outbreak, which could indicate contamination with wastewaters. The majority of samples belonged to GII.3[P12], followed by GII.2[P16], GII.17[P17], and GII.4 Sydney[P16]. We next proceeded with whole-genome sequencing and obtained full genomes for 19 samples. Phylogenetic analysis revealed that some of the isolates showed high similarity with the sequences isolated from the United States related to the same outbreak. We also analyzed amino acid variations in the sequenced genomes and found that overall the GII.3[P12] isolates have lower variations compared to other genotypes.

**IMPORTANCE:**

Norovirus outbreaks associated with contaminated shellfish occur frequently. Whole-genome sequencing (WGS) could play a critical role in understanding and controlling norovirus outbreaks as it allows for source attribution, tracking transmission pathways, and detecting recurrent or linked outbreaks. Here, we described how the data obtained by WGS were employed for understanding transmission patterns and norovirus epidemiology.

## INTRODUCTION

Noroviruses are the most common cause of acute gastroenteritis, leading to significant morbidity and mortality in children, the elderly, and immunocompromised individuals (1, 2), with an estimated economic burden of $10.6 billion per year, in the United States (3). Despite some promising trials, currently, no vaccine or antivirals are approved to reduce the burden of noroviruses (3). Viral transmission usually occurs through the fecal-oral route or through contaminated food and surfaces (4)

Noroviruses are non-enveloped icosahedral particles, which belong to the *Caliciviridae* family (5). Their genome is an approximately 7.5 kb, positive-sense, single-stranded RNA that encodes three open reading frames (ORFs). ORF1 encodes a polyprotein that is cleaved into six non-structural viral proteins, including the RNA-dependent RNA polymerase (RdRp). ORF2 encodes VP1, the major structural capsid protein, and ORF3 encodes VP2, a minor structural capsid protein (5). Norovirus genomes are highly diverse due to the accumulation of point mutations and recombination (6, 7). To date, noroviruses are classified into at least 10 genogroups and at least 49 genotypes based on the diversity of the 5′ ORF2 end and 60 types based on the diversity of the RdRP gene (NS7) (8). Therefore, for outbreak investigations and epidemiological surveillance of noroviruses, both ORF1 and ORF2 are typed. Currently, whole-genome sequencing (WGS) is becoming more common for viral outbreak investigations (9). It is also hypothesized that the success of vaccine trials and antivirals could be dependent on the viral genotype (10). Thus, genomic data could be employed for outbreak investigations, source tracking, and vaccine and antiviral designs.

Norovirus is estimated to be responsible for 48% of all shellfish-associated outbreaks, which can result in secondary transmission with rates potentially as high as 88% (11–13). Bivalved molluscan shellfish present a relatively high risk of being implicated in norovirus transmissions due to their ability to concentrate viruses from contaminated water and being consumed raw or undercooked (14–16). In January to April 2022, a multi-jurisdictional norovirus outbreak associated with contaminated oysters from British Columbia (BC) led to multiple recalls and hundreds of human illnesses (17). In this study, we performed genomic analysis on 30 clinical samples obtained from this norovirus outbreak. Furthermore, we conducted phylogenetic, intra-host nucleotide diversity and amino acid mutation analyses on the linked genomes.

## RESULTS

### Epidemiological and molecular analysis

From January to April 2022, contaminated oysters from BC were attributed to over 339 norovirus infections in five provinces in Canada (17) and 192 illnesses in 13 states in the US (18). Our lab received 30 clinical samples associated with the outbreak from two provinces. As indicated in Table 1, 29 samples were positive for norovirus genogroup 2 (GII) and one sample positive for norovirus genogroup 1 (GI). The majority of the samples were from Saskatchewan. The most common genotype was GII.3 (*n* = 13), followed by GII.2 (*n* = 6), GII.4 (*n* = 3), GII.6 (*n* = 3), GII.17 (*n* = 3), and GII.22 (*n* = 2; Table 1). Viral titers varied from 4 × 10^6^ to 2.4 × 10^1^ genome copies (gc) per microliter of the extracted RNA.

**TABLE 1:** Samples received for diagnosis, their titers, capsid genotypes, and amplicon sizes

Sample ID	Province	Titer (genome copies/µL)	Genotype	Band size
BMH-146	SK^*a*^	2.61E + 03	GII.3	7 and 5 kb
BMH-147	SK	4.29E + 01	GII.17	4 kb
BMH-148	SK	1.16E + 04	GII.3	7 and 2.5 kb
BMH-149	SK	6.67E + 04	GII.3	7 kb
BMH-150	SK	3.96E + 06	GII.3	7 kb
BMH-151	SK	9.75E + 02	GII.4	4 kb
BMH-152	SK	1.64E + 04	GII.6	7 and 2 kb
BMH-153	SK	1.37E + 06	GII.4	7, 4, 2, and 1 kb
BMH-154	SK	1.74E + 02	GII.2	7, 6, 5, and 4 kb
BMH-155	SK	4.46E + 01	GII.17	3.5 kb
BMH-156	SK	2.55E + 05	GII.2	7, 3.5, 2.6, 1.8, and 1.6 kb
BMH-157	SK	4.12E + 03	GII.3	1.3 kb
BMH-158	SK	1.88E + 05	GII.22	7, 3, and 2.5 kb
BMH-159	SK	2.35E + 07	GII.3	7, 4, 3.5, 3, 2, and 1.8 kb
BMH-160	SK	2.44E + 01	GII.6	No band
BMH-161	SK	4.75E + 01	GII.6	No band
BMH-162	SK	7.13E + 01	GII.4	No band
BMH-163	SK	1.71E + 05	GII.3	No band
BMH-164	MB	3.24E + 05	GII.2	7, 3, 2.6, 1.6, and 1.3 kb
BMH-165	MB	1.20E + 05	GII.2	7, 4, 3, 2.6, 1.8, and 1.5 kb
BMH-166	MB	1.15E + 05	GII.2	7 and 3 kb
BMH-167	SK	1.09E + 04	GII.3	No band
BMH-168	SK	7.63E + 03	GII.17	7 kb
BMH-169	SK	2.50E + 04	GII.3	7 and 3 kb
BMH-170	SK	9.54E + 02	GII.3	Not done
BMH-171	SK	1.96E + 06	GII.3	3 kb
BMH-172	SK	1.66E + 04	GI.7	Not done
BMH-173	SK	2.02E + 05	GII.2	2.6, 1.8, 1.5, and 1.4 kb
BMH-174	SK	5.89E + 05	GII.22	2.5 kb
BMH-175	SK	9.22E + 03	GII.3	7 and 3 kb

^*a*^ SK, Saskatchewan.

### WGS analysis

To further investigate the epidemiological links between these samples, we performed full-genome analysis using the methods that have been described previously for all of the GII-positive samples (6, 19). Full-size amplicons were obtained for 16 samples, partial amplicons were achieved for 7 samples, and 5 samples did not produce any sequenceable amplicons likely due to low viral titer in the sample (19) (Table 1). Near full genomes were assembled for 19 samples that were then subjected to further analysis (Table 2). No evidence of co-infection of patient samples was identified by WGS.

**TABLE 2:** Genome sequencing and assembly metrics of 19 norovirus GII samples from the outbreak

Sample	Total reads	% Reads norovirus	% Reads human	Average coverage depth (×)	Assembled genome size (bp)
BMH-22–146	225,178	24.7	15.9	6,919	7,614
BMH-22–148	55,240	2	5.1	29	7,415
BMH-22–149	84,424	30	8.3	951	7,577
BMH-22–150	165,494	64.6	1.2	3,612	7,577
BMH-22–151	7,047,126	91.9	0	240,959	7,547
BMH-22–152	3,041,114	74.9	0	103,984	7,556
BMH-22–153	123,282	94.3	3	2,604	7,657
BMH-22–154	63,478	78.9	7.8	1,134	7,642
BMH-22–156	1,135,922	92.8	0	41,823	7,531
BMH-22–158	1,123,554	97.8	0	43,750	6,394
BMH-22–159	413,034	31.8	0.1	11,699	7,522
BMH-22–164	124,850	97.9	0	4,092	7,714
BMH-22–165	61,556	99	0	2,290	7,526
BMH-22–166	1,551,470	97.7	0	56,910	7,533
BMH-22–168	280,476	98.3	0	9,645	7,650
BMH-22–169	82,790	27.9	0	2,843	7,616
BMH-22–171	61,200	27.7	0	2,126	7,373
BMH-22–173	22,054	28.7	64	231	7,520
BMH-22–175	275,094	12.9	22	4,872	7,640

### Phylogenetic analysis

**Fig 1:**
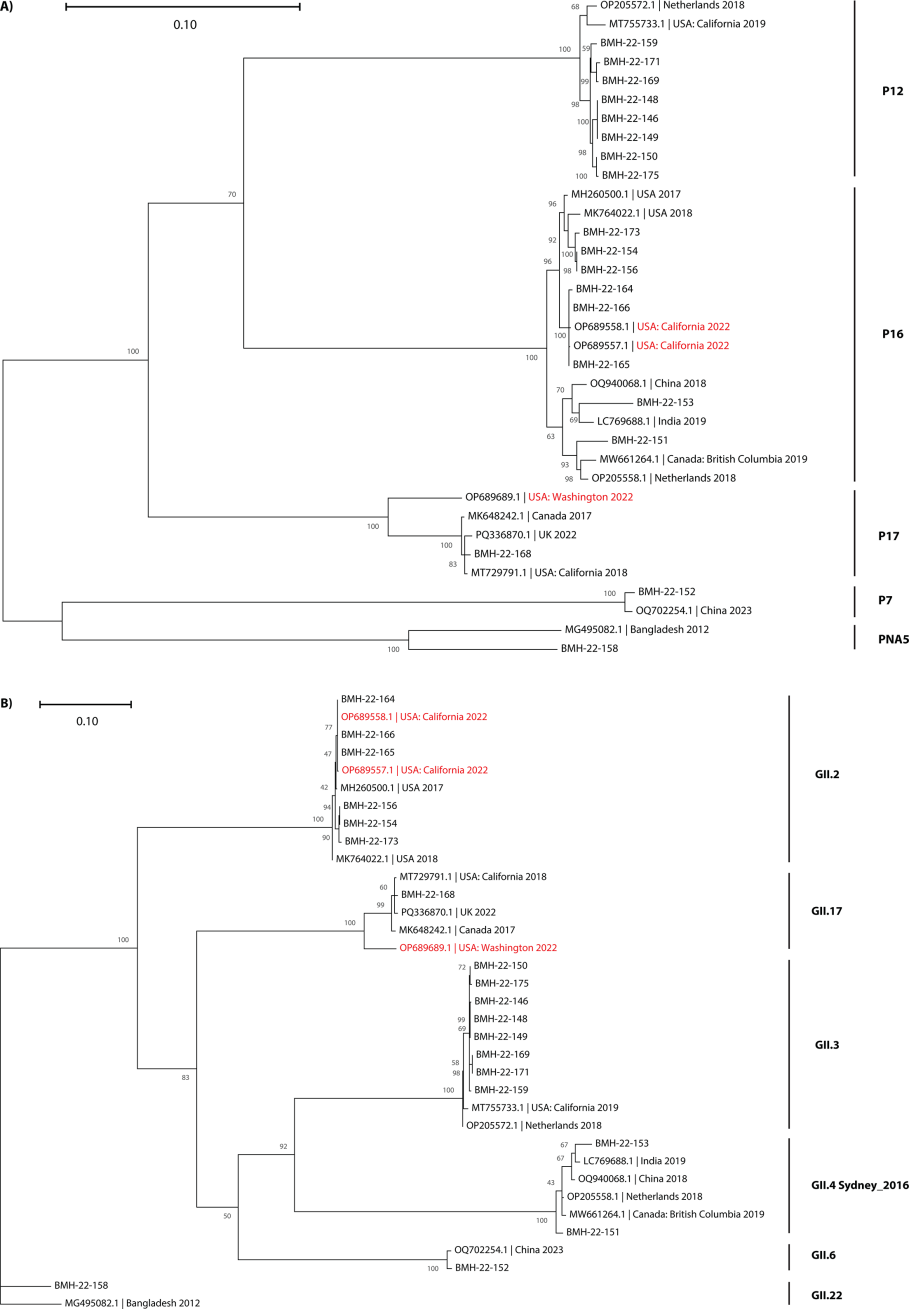
Phylogenetic analysis of norovirus GII sequences from the BC oyster outbreak in 2022. Maximum Likelihood trees for (**A**) ORF1 and (**B**) ORF2. Trees were constructed using MEGA (v11.0.13) and 1,000 bootstrap replicates. The scale bars represent the phylogenetic distance expressed as nucleotide substitutions per site. Bootstrap values are shown at each node. Reference sequences were obtained from GenBank with US isolates associated with the BC outbreak shown in red. Genotypes of all isolates are shown on the right.

As demonstrated in Fig. 1A and B, the phylogenetic analysis from the WGS data is consistent with the genotyping performed by Sanger sequencing (Table 1). Interestingly, our sequences from GII.2[P16] clustered closely with two sequences from GenBank (OP689557 and OP689558) that were related to the same outbreak but were isolated from patients in California. These two isolates showed 99.9% identity by BLAST for ORF1 and ORF2 to BMH-22–164, BMH-22–165, and BMH-22–166. They also showed high similarity with the rest of the GII.2[P16] isolates (>98.7% for ORF1 and >99.1% for ORF2). The GII.17[P17] BMH-22–168 sequence, however, only showed 93% identity for ORF1 and ORF2 with OP689689, which was also associated with the same oyster outbreak from Washington State. Thus, BMH-22–168 might not be directly related to the 2022 outbreak case from Washington but was closely related to GII.17[P17] isolates from the UK 2022, California 2018, and Canada 2017 (PQ336870, MT729791, and MK648242).

As mentioned, the majority of sequences belong to GII.3[P12], which clustered closely with each other without major genetic variety. The closest related isolates for ORF1 (98%) and ORF2 (99%) were from California 2019 (MT755733) and Netherlands 2018 (OP205572). Finally, there was a cluster of GII.4Sydney[P16], which is a commonly identified strain, a GII.6[P7] isolate, and a GII.22[PNA5] isolate, which is rarely reported (Fig. 1A and B). Interestingly, no closely related isolates for BMH-22–158, GII.22[PNA5], could be identified in GenBank (88% for ORF1 and ORF2).

### Intra-sample nucleotide diversity and amino acid variant analysis

**Fig 2:**
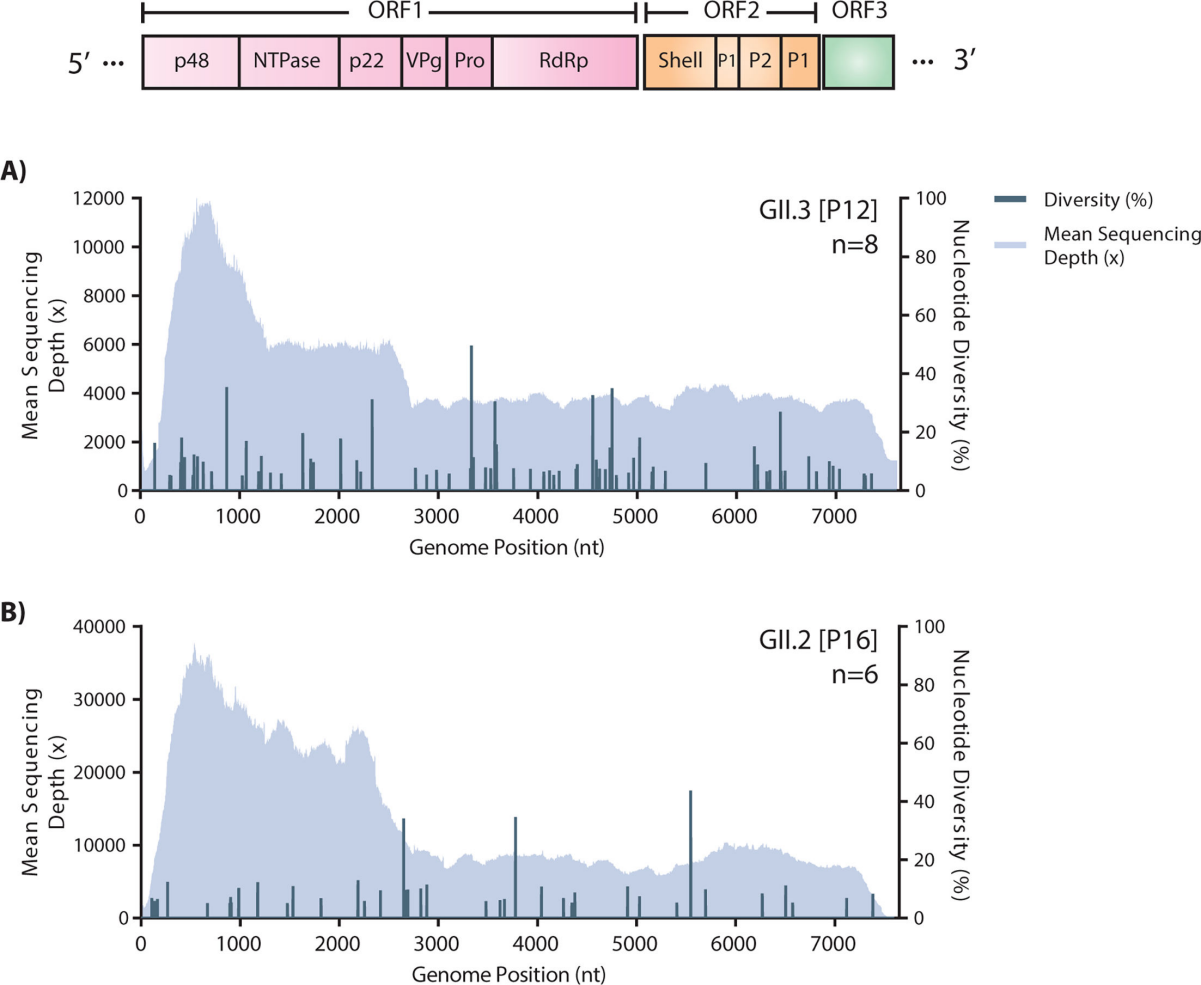
Intra-sample nucleotide diversity of norovirus genomes. Mean sequencing depth (mapped reads at each nucleotide position) and nucleotide diversity (%) along the genomes are shown for each genotype. Nucleotide diversity >5% with at least 100 reads at each position is included. Schematic representation of the norovirus genome is depicted (top).

To gain insight into the genetic variation of the oyster isolates, full sequences from the most common genotypes, GII.3 [P12] and GII.2 [P16], were assessed for intra-sample nucleotide diversity (Fig. 2; Table 3). Nucleotide variants at a position were considered present if observed in at least 5% of the sequenced reads. Overall, variants were observed throughout the norovirus genomes. Furthermore, per genotype, a similar number of average nucleotide variants of 9.6 (ranges 1–15) and 6.3 (ranges 0–20) were observed for the GII.3 [P12] and GII.2 [P16] genomes, respectively. These results demonstrate the presence of minor genetic diversity within a single host for these genotypes, consistent with reported levels of variants observed during acute norovirus infections (20, 21). Of note, the mean sequencing depth for each genotype was greater at the 5′ end of the sequences (Fig. 2).

**TABLE 3:** Nucleotide variants identified in norovirus samples by WGS

Sample	Genotype	Total reads	Nucleotide variants (#)	% Nucleotide variants/reads
BMH-22–146	GII.3[P12]	225,178	15	6.66 × 10^−03^
BMH-22–148	GII.3[P12]	55,240	3	5.43 × 10^−03^
BMH-22–149	GII.3[P12]	84,424	15	1.78 × 10^−02^
BMH-22–150	GII.3[P12]	165,494	1	6.00 × 10^−04^
BMH-22–151	GII.4[P16]	7,047,126	1	1.00 × 10^−05^
BMH-22–152	GII.6[P7]	3,041,114	13	4.27 × 10^−04^
BMH-22–153	GII.4[P16]	123,282	1	8.10 × 10^−04^
BMH-22–154	GII.2[P16]	63,478	6	9.45 × 10^−03^
BMH-22–156	GII.2[P16]	1,135,922	0	0
BMH-22–158	GII.22[PNA5]	1,123,554	214	1.91 × 10^−02^
BMH-22–159	GII.3[P12]	413,034	12	2.91 × 10^−03^
BMH-22–164	GII.2[P16]	124,850	4	3.20 × 10^−03^
BMH-22–165	GII.2[P16]	61,556	3	4.87 × 10^−03^
BMH-22–166	GII.2[P16]	1,551,470	5	3.20 × 10^−04^
BMH-22–168	GII.17[P17]	280,476	6	2.14 × 10^−03^
BMH-22–169	GII.3[P12]	82,790	7	8.46 × 10^−03^
BMH-22–171	GII.3[P12]	61,200	11	1.80 × 10^−02^
BMH-22–173	GII.2[P16]	22,054	20	9.07 × 10^−02^
BMH-22–175	GII.3[P12]	275,094	13	4.73 × 10^−03^

Amino acid variants of the sequences from the same genotypes relative to reference sequences are shown in Table 4 for GII.2[P16] and Table 5 for GII.3[P12]. Multiple amino acid variations were observed in P48 of ORF1 for both P16 and P12 isolates (Tables 4 and 5). As expected, within ORF2, P2 had the highest number of single nucleotide polymorphisms (SNPs) in both GII.2 and GII.3 genotypes (Tables 4 and 5). Importantly, we observed that the linked sequences do not show much variation, and many of the amino acid changes are biochemically conservative such as V→I, L→Y, K→R, and G→E, further highlighting the high similarity of the linked isolates.

**TABLE 4:** Comparison of amino acid variations in Norovirus GII.2 [P16] isolates from this study^*f*^

	ORF1													ORF2							
Sample	P48^*a*^			NTPase		P22		Vpg^*b*^	Pro^*c*^	RdRp				N^*d*^	Shell	P1^*e*^	P2				P1
GII.2 [P16]	161	227	305	488	669	795	857		1,077	1,244	1,310	1,463	1,613				335	348	389–390	396	448
OP689557	V	V	L	I	K	A	V		T	S	H	I	E				V	N	VK	L	N
BMH-22–164	V	V	L	I	K	A	V		T	S	H	I	E				V	N	VK	L	N
BMH-22–165	V	V	L	I	K	A	V		T	S	H	I	E				V	N	VK	L	N
BMH-22–166	V	V	L	I	K	A	V		T	S	H	I	E				V	N	VK	L	N
BMH-22–173	I	I	F	M	R	A	A		A	G	H	T	G				I	S	VR	F	S
BMH-22–156	I	I	F	I	R	T	A		A	G	Y	T	E				I	S	AR	L	N
BMH-22–154	I	I	F	I	R	T	A		A	G	Y	T	E				I	S	AR	L	N

^*a*^ N-terminal protein.

^*b*^ Viral protein.

^*c*^ Protease.

^*d*^ Short N-terminal arm.

^*e*^ Protruding domain.

^*f*^ ORF1 and ORF2 coding regions and amino acid positions relative to GenBank Norovirus reference strain CA-RGDS-1162 (accession OP689557) are shown.

**TABLE 5:** Comparison of amino acid variations in Norovirus GII.3 [P12] isolates from this study^*f*^

	ORF 1													ORF2				
Sample	P48^*a*^					NTPase	P22				Vpg^*b*^	Pro^*c*^	RdRp	N^*d*^	Shell	P1^*e*^	P2	P1
GII.3 [P12]	44	52	85	186	232	398	737	775	782	846	1,006		1,409		102	263	295	
MN416945	na	na	na	na	F	N	V	K	A	D	N		N		G	N	S	
BMH-22–169	Q	I	V	D	Y	N	V	K	A	D	N		N		G	N	S	
BMH-22–171	na	na	V	D	Y	N	V	K	A	D	N		S		G	N	S	
BMH-22–159	Q	I	A	D	F	N	I	K	A	D	N		N		G	N	N	
BMH-22–150	H	V	A	D	F	S	V	R	A	D	N		N		G	N	S	
BMH-22–175	H	V	A	D	F	S	V	R	A	D	H		N		G	S	S	
BMH-22–148	na	na	A	G	F	N	V	K	T	G	N		N		G	N	S	
BMH-22–146	Q	V	A	G	F	N	V	K	T	G	N		N		E	N	S	
BMH-22–149	Q	V	A	G	F	N	V	K	T	G	N		N		G	N	S	

^*a*^ N-terminal protein.

^*b*^ Viral protein.

^*c*^ Protease.

^*d*^ Short N-terminal arm.

^*e*^ Protruding domain; na, no sequence data available at nucleotide position.

^*f*^ ORF1 and ORF2 coding regions and amino acid positions relative to GenBank Norovirus reference strain G19_047 (accession MN416945) are shown.

## DISCUSSION

In oyster-related norovirus outbreaks, multiple virus strains have frequently been observed both in infected patients and in the corresponding oysters (12, 22), and typically when multiple genotypes are detected, sewage contamination is thought to be the cause (12).

Although the partial sequences obtained by dual typing are informative, they do not provide enough resolution for understanding transmission chains and viral evolution. Performing WGS on next-generation sequencing platforms allows for the identification of single nucleotide variants and thereby enables differentiation between genotypically similar strains (23, 24). For this reason, norovirus WGS is increasingly being applied for outbreak investigations (9). We have previously employed an amplicon system developed by Parra and colleagues (6) to conduct an in-depth genomic analysis of norovirus GII.4 strains isolated in Canada (19). Performing WGS of noroviruses may help with the classification of norovirus and, during outbreak investigations, could be helpful for the scientific community and regulatory authorities in further understanding causes and transmission chains.

The predominant genotype identified in this outbreak was GII.3[P12], followed by GII.2[P16], GII.17[P17], and GII.4 Sydney[P16]. Unfortunately, there is no data on the prevalent norovirus genotypes in BC in clinical samples, wastewaters, or the oyster harvest sites prior or during this outbreak. However, Calicinet surveillance data reveal that the dominant genotypes in this study were among the commonly identified norovirus genotypes in outbreaks in the USA in 2022–2023 (https://www.cdc.gov/norovirus/php/reporting/calicinet-data.html). This observation further confirms that a point source could not be responsible for the oyster contamination, and a broader source such as wastewater could be suspected as multiple norovirus genotypes were identified. Interestingly, we found closely related sequences to GII.2[P16] and GII.17[P17] in GenBank associated with the same outbreak from patients residing in the USA, which further confirms the epidemiological data on the source of the illnesses.

Sequence diversity was observed between GII.2[P16] and GII.4 Sydney[P16]; however, GII.3[P12] sequences were mostly similar, and this could be deduced from both phylogenetic and SNP analyses. This information along with the epidemiological data could link otherwise geographically separated cases to a food source and is therefore another indication that WGS analysis of noroviruses could be considered for outbreak investigations.

A major limitation of this study was the lack of data from the oysters that were implicated in the outbreak as we did not have access to those samples. Therefore, the epidemiological studies were limited to the clinical samples that were received for diagnostic purposes. Furthermore, only a limited number of fecal samples were analyzed in this study. Lack of access to multiple clinical and food samples in an outbreak investigation is common. Several challenges hinder the collection of fecal specimens during foodborne outbreaks. These include patients’ perception that providing stool samples is unnecessary after recovery, privacy concerns, insufficient personnel dedicated to specimen collection, and logistical difficulties related to sample transportation (25). Nevertheless, we were able to analyze 30 clinical samples from this outbreak and conducted post-outbreak epidemiological studies. In the future, it would be beneficial to extend the analysis beyond food and clinical samples to include testing of water from shellfish growing and relaying areas, providing deeper insight into the dynamics of shellfish-associated outbreaks. Furthermore, as highlighted by our phylogenetic analysis, there is also a need for a harmonized and accessible database, such as GenBank, to provide researchers with access to additional norovirus sequences that could be relevant to outbreak investigations.

In conclusion, this study demonstrated the utility of a WGS for the testing of clinical samples in a norovirus outbreak investigation. We also highlighted how food exportation may contribute to the spread and exchange of viral strains between countries.

## MATERIALS AND METHODS

### Clinical sample collection and preparation

In March and April 2022, our lab received 30 stool samples from Manitoba and Saskatchewan public health laboratories associated with the norovirus oyster outbreak (Table 1). The presence of norovirus RNA was confirmed by droplet digital PCR (Bio-Rad, Mississauga, Ontario, Canada) using the probes and primers that were described previously (23, 24). This study was granted an exemption from aquiring ethics approval by Health Canada, and formal consent was not required because of the consistent research use and anonymization of the samples.

### RNA extraction and titer determination

RNA extraction was performed as described previously (19) using the MagMax Viral RNA Isolation Kit (Ambion) following the manufacturer’s recommendations. Viral titers were determined by droplet digital PCR (Bio-Rad, Mississauga, Ontario, Canada) using the probes and primers that were described previously (23, 24).

### Sanger sequencing

Positive norovirus stool samples were genotyped by Sanger sequencing. For this purpose, conventional reverse transcription PCR (RT-PCR) was carried out targeting a 330 bp region in ORF2, the 5′-end of the major capsid protein VP1 (26). For four samples that were unsuccessful by conventional RT-PCR for the ORF2, we amplified a 213 bp region in the RdRp of the ORF1 (27).

### Full-length amplicon generation and Illumina sequencing

Amplification of the full-length genome was performed as described previously (19) using a set of primers that target the conserved regions of the 5′- and 3′-ends of GII noroviruses (GII1-35: GTGAATGAAGATGGCGTCTAACGACGCTTCCGCTG, and Tx30SXN) and the SequalPrep Long PCR Kit (Invitrogen) following the manufacturer’s recommendations.

Illumina norovirus libraries were constructed using the NexteraXT DNA Library Preparation Kit and Nextera DNA UD Indexes according to the manufacturer’s instructions (Illumina Inc.). Paired-end Illumina sequencing was performed on a MiSeq instrument (v3 chemistry, 2 × 300 bp) according to manufacturer instructions (Illumina Inc.).

### Read processing, *de novo* genome assembly, and genotyping

Raw Illumina reads were processed using FastP (v0.20.1) to remove adapter and barcode sequences, correct mismatched bases in overlaps, and filter low-quality reads (Q < 20) (28). *De novo* assemblies for norovirus strains were generated using Megahit (v 1.2.9) using default settings (29). Filtered reads and contigs were taxonomically classified using Kraken2 (v2.1.3) and the standard Kraken2 database (NCBI RefSeq bacterial, archaeal, viral, human, and vector database downloaded 19 January 2024) (30). Norovirus contigs were extracted, and error correction was performed with Polypolish v0.4.3 (31) and Polca v4.0.5 (github.com/alekseyzimin/masurca/releases). The norovirus genomes were genotyped using the Norovirus Genotyping Tool v2.0 (32).

### Coverage plots

Illumina reads were mapped to the norovirus *de novo* whole genomes using BBMap (v38.18, sourceforge.net/projects/bbmap). Depth of coverage was assessed using the Samtools depth function (v1.17, github.com/samtools/samtools), and data were graphed using GraphPad (v6.01).

### Amino acid variant analysis and intra-sample nucleotide variant analysis

Amino acid variants were identified for each norovirus sample using MUSCLE within MEGA (v11.0.13) (33) for alignment. Variants were identified using the GenBank reference genomes OP689557 and MN416945 for the GII.2[P16] and GII.3[P12] genotypes, respectively. Intra-sample nucleotide variants were identified by Breseq (v0.38.1) (34) using the polymorphism prediction pipeline, polymorphism-frequency-cutoff of 0.05, and Illumina reads and respective consensus assemblies for each isolate. Nucleotide variant inclusion criteria consisted of at least 100 reads and greater than 5% diversity at the nucleotide position.

### Phylogenetic analysis

ORF1 and ORF2 nucleotide sequences from norovirus isolates from this study and GenBank reference sequences were aligned with MUSCLE using MEGA (v11.0.13). Maximum likelihood phylogenetic trees based on the Tamura–Nei model were constructed and visualized in MEGA using the aligned sequences and 1,000 bootstrap replicates.

## Data Availability

The *de novo* genome sequences of the 19 norovirus isolates used in this study have been uploaded to the GenBank under accession numbers: PQ273182 to PQ273200. All SRAs are available in the GenBank under BioProject ID PRJNA1140557.
